# Graphene-Templated Achiral Hybrid Perovskite for Circularly Polarized Light Sensing

**DOI:** 10.1021/acsami.4c10289

**Published:** 2024-09-19

**Authors:** Oleksandr Volochanskyi, Golam Haider, Essa A. Alharbi, George Kakavelakis, Martin Mergl, Mukesh Kumar Thakur, Anurag Krishna, Michael Graetzel, Martin Kalbáč

**Affiliations:** † Department of Low-dimensional Systems, J. Heyrovsky Institute of Physical Chemistry of the Czech Academy of Sciences, Dolejšková 2155/3, 18223 Prague, Czech Republic; ‡ Faculty of Chemical Engineering, Department of Physical Chemistry, University of Chemistry and Technology in Prague, Technická 5, 14200 Prague, Czech Republic; § Microelectronics and Semiconductors Institute, King Abdulaziz City for Science and Technology (KACST), Riyadh 11442, Saudi Arabia; ∥ École Polytechnique Fedérale du Lausanne, Laboratory of Photonics and Interfaces, Station 6, Lausanne 1015, Switzerland; ⊥ Department of Electronic Engineering, School of Engineering, Hellenic Mediterranean University, Romanou 3, Chalepa, GR-73100 Chania, Crete, Greece

**Keywords:** chirality, optical helicity sensing, Rashba splitting, graphene, perovskite, photodetector

## Abstract

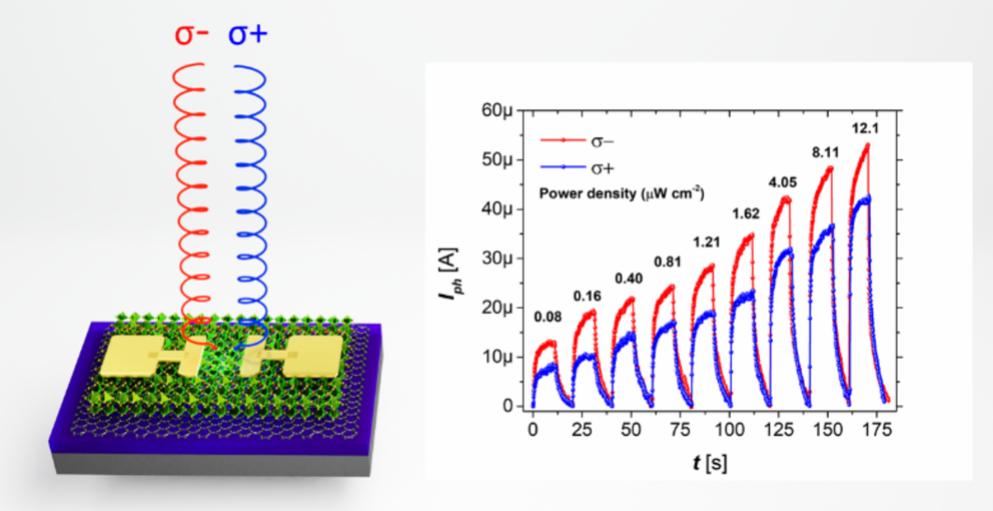

This study points out the importance of the templating effect in hybrid organic–inorganic perovskite semiconductors grown on graphene. By combining two achiral materials, we report the formation of a chiral composite heterostructure with electronic band splitting. The effect is observed through circularly polarized light emission and detection in a graphene/α–CH(NH_2_)_2_PbI_3_ perovskite composite, at ambient temperature and without a magnetic field. We exploit the spin–charge conversion by introducing an unbalanced spin population through polarized light that gives rise to a spin photoconductive effect rationalized by Rashba-type coupling. The prepared composite heterostructure exhibits a circularly polarized photoluminescence anisotropy *g*_CPL_ of ∼0.35 at ∼2.54 × 10^3^ W cm^–2^ confocal power density of 532 nm excitation. A carefully engineered interface between the graphene and the perovskite thin film enhances the Rashba field and generates the built-in electric field responsible for photocurrent, yielding a photoresponsivity of ∼10^5^ A W^–1^ under ∼0.08 μW cm^–2^ fluence of visible light photons. The maximum photocurrent anisotropy factor *g*_ph_ is ∼0.51 under ∼0.16 μW cm^–2^ irradiance. The work sheds light on the photophysical properties of graphene/perovskite composite heterostructures, finding them to be a promising candidate for developing miniaturized spin-photonic devices.

## Introduction

The spin degree of freedom and its manipulation has become a hot research topic across the material sciences, with the goal of developing next-generation photonic, opto-spintronic, and nonvolatile memory devices.^1^ Optically generated spin polarization is the most common approach to investigate the practical information capacity of light and polarization-related effects in electronic circuits. In current technology, circularly polarized light (CPL) finds widespread applications, including in circular dichroism spectroscopy,^2^ imaging,^3^ holography,^4^ tomography,^5^ and ellipsometry.^6^ However, it can also be found at the heart of many novel photonic technologies, including spin-based optical communication,^7,8^ quantum-based computing,^9,10^ information transmission, processing, and storage,^11,12^ and polarization-probe polarization-imaging techniques, including LiDAR.^13^ Commercial devices typically employ polarization-insensitive photosensors combined with bulky optical components. Such designs are expensive and difficult to miniaturize. Therefore, developing more efficient methods of manipulating and detecting light polarization can lead to improvements to conventional devices, but can also lead to new types of polarization-sensitive photonic devices essential for future technological development.

As a rising star material with remarkable electrical properties and a unique band structure, single-layer graphene (SLG) shows strong potential in diverse fields, including optoelectronic technology. Owing to its gapless band structure, graphene absorbs a broad range of wavelengths (from UV to IR) and then ballistically conducts the photoexcited carriers.^14,15^ Graphene’s short exciton lifetime and absence of intrinsic gain mechanism, makes direct use of pristine graphene photodetectors difficult.^16,17^ However, these problems can be overcome by introducing semiconducting light absorbers, which can prevent fast exciton recombination and enable gain-supported photodetection. Although high responsivity and gain in graphene-based photodetectors have already been demonstrated,^18,19^ these devices can be given substantial added value, such as polarized light sensitivity, available from graphene/light absorber interface engineering, as demonstrated in this study.

A straightforward way to achieve CPL sensitivity is application of chiral organic molecules^20−22^ or other anisotropic media, such as chiral (plasmonic) metasurfaces.^4,23^ In addition, the combination of chiral molecules and confinement effects due to reduced dimensionality (0D quantum dots, 1D nanowires, and 2D layers) can offer enhanced CPL sensitivity. As a result, low-dimensional chiral perovskites have emerged as suitable anisotropic media. Several reports have been published on circularly polarized sensors and emitters based on chiral perovskites of different dimensionality.^24−28^ While such an approach can yield anisotropy factors on the order of 10^–1^, the resulting devices usually suffer from poor optoelectronic performance. For instance, devices made solely from chiral perovskite materials typically exhibit a room-temperature responsivity no higher than ∼10^1^ A W^–1^.^28,29^

Among perovskite materials, hybrid organic–inorganic perovskites (HOIPs), such as the black α-phase formamidinium (CH(NH_2_)_2_, hereafter FA) lead iodide (α-FAPbI_3_), have become one of the most promising candidates for high-efficiency solar cells due to their crystal lattice properties and relative stability under ambient conditions.^30,31^ Although HOIPs have been studied for decades, only recently has the significance of spin–orbit coupling (SOC) for optoelectronics been emphasized, first based on DFT calculations and then confirmed experimentally.^32,33^ The presence of heavy elements (Pb, X, where X is halogenide) in the HOIP lattice introduces strong SOC in the electronic structure, determining the shape of electronic bands near their extrema points. The spin–orbit interaction couples spin degrees of freedom with electronic orbits, thereby offering a beneficial communication channel between the spin space and the crystal lattice. The spin-flip effect in the absence of a magnetic field was described in 1955,^34^ and has since received a great deal of interest, as the manipulation of an electron’s spin instead of its charge is highly desirable. The absence of inversion symmetry, as observed in bulk noncentrosymmetric crystals or at the interface of centrosymmetric crystals, combined with the presence of the SOC effect, gives rise to an effective magnetic field within the crystal lattice. This field is responsible for a spin-dependent shift of the electronic bands along the *k* direction in the momentum space, resulting in a splitting of otherwise spin-degenerate bands, a phenomenon referred to as Rashba–Dresselhaus SOC.^34−37^ Consequently, the band gap becomes slightly indirect, allowing spin-dependent optical transitions to occur.^38^ Generally, the prerequisite of inversion asymmetry is satisfied by ferroelectrics, which can be treated as bulk Dresselhaus-type SOC material. On the other hand, Rashba–Edelstein SOC arises from structural site inversion asymmetry, for example, at the surfaces or heterojunctions.^39^

Although spontaneous polarization and ferroelectricity signatures have been demonstrated in 2D HOIPs,^35,40,41^ the origin of inversion asymmetry in 3D or mixed 2*D*/3D HOIPs remains unclear and controversial as it necessitates the coexistence of centrosymmetry and ferroelectricity. The incorporation of chiral organic molecules into HOIPs brings spin-filter-like properties due to chirality-induced spin selectivity. Therefore, the CPL sensitivity can be attributed to phenomena induced by dipolar interactions with polarizable π clouds of chiral organic cations resulting in organic-to-inorganic chirality transfer.^24,25,42^ Various studies have suggested that Rashba splitting in achiral perovskites might result from local ferroelectric phase formation during the gradual degradation of the cubic phase.^36,40^ Others have pointed out a considerable rotational motion of the A-site cations on the picosecond time scale, which may result in local breaking of the inversion symmetry and lead to so-called dynamic Rashba splitting.^43^ Some other reports have emphasized the role of the ionic defects present in 3D HOIPs, and have related the occasionally observed Rashba effect to the density of such defects in the bulk crystal lattice, which ultimately depends on the fabrication procedure.^44^

Despite importance, a little consideration has been given to the fact that the growth of solution-processed HOIPs is also substrate-templated.^45,46^ For instance, theoretical studies have indicated that during the formation of HOIP films on graphene, lead halide octahedra experience an intrinsic ferroelectric displacement leading to naturally distorted states at the graphene/perovskite interface.^47−49^ More precisely, the interaction of lead halide-octahedra-terminated states induces electronic charge density redistribution at the graphene/perovskite interstitial region during growth. This redistribution consequently suppresses octahedral tilt, which further impairs the centrosymmetric nature of the lead halide at the interface. The charge redistribution also generates a surface junction (i.e., a built-in electric field) that additionally promotes the separation of electrons and holes, producing a depletion region. We presume that such interfacial ferroelectric distortions, resulting from the octahedral connectivity of HOIPs to the graphene, might explain the origin of the CPL-related effects and apparent Rashba splitting in graphene/hybrid perovskite composites. In the absence of inversion asymmetry, the direction of the photocurrent does not depend on the helicity of the incoming light. Thus, a neat way to probe Rashba-type couplings in a heterojunction is to exploit the spin–charge conversion through CPL, leading to a family of spin photoconductive effects (SPEs) such as the circular photogalvanic effect and the spin-galvanic effect.^41^

In this vein, we bring together solution-processed α-FAPbI_3_ perovskite—a strong light absorber with a potential for strong Rashba SOC—and chemical vapor deposited (CVD) graphene as a primary hole conductor. By combining these two nonchiral materials we obtain a remarkable result—a composite with chiral properties. We probe its band structure by applying photoluminescence analysis and further demonstrate how left- and right-handed excitations provide an indication for phototransport in spin-split bands that can be used for CPL detection.

## Results and Discussion

**Figure 1:**
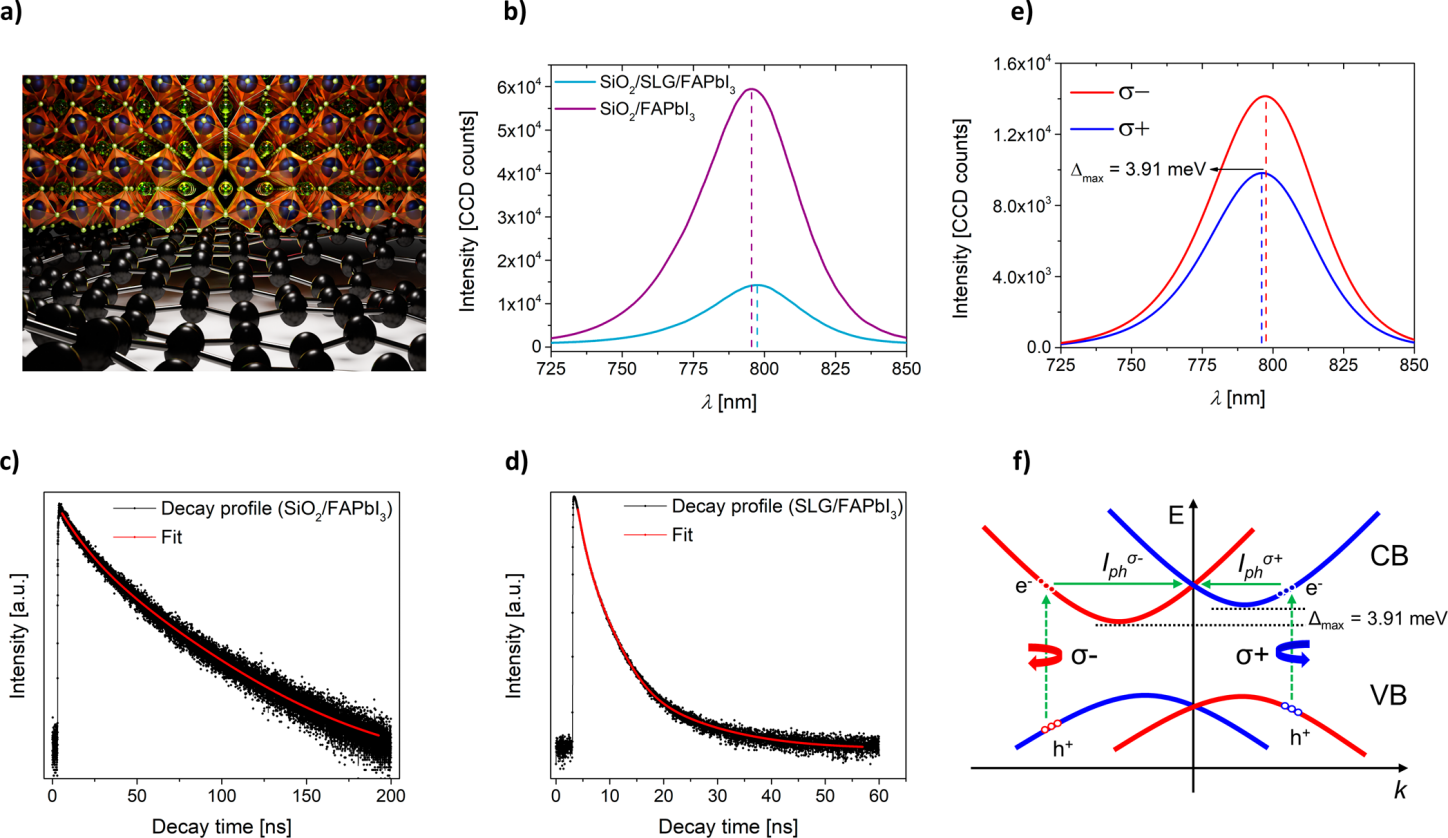
Spectroscopic characterization of the device. (a) An illustrative schematic of the exaggerated octahedral distortions in the perovskite lattice due to interactions with graphene at the interface. Note: This schematic is not to scale and is intended to demonstrate potential structural effects, not the exact atomic arrangement. (b) A comparison of PL spectra collected on graphene/α-FAPbI_3_ perovskite composite (light blue) and α-FAPbI_3_ perovskite deposited on SiO_2_ substrate (purple). The emission maxima on graphene/α-FAPbI_3_ and SiO_2_/α-FAPbI_3_ at 798 and 794 nm, respectively. (c), (d) TCSPC decay profiles collected on perovskite on SiO_2_ and graphene/perovskite composite, respectively. (e) Average of circularly polarized PL spectra collected on graphene/α-FAPbI_3_ composite. The data were collected in a confocal regime within the gap of the device using 2.54 × 10^3^ W cm^–2^ power density of 532 nm excitation. (f) A schematic representation of Rashba splitting in HOIP and spin-polarized photocurrent (*I*_ph_) generation. The direction of the *I*_ph_ (solid arrows) depends on the helicity of excitation (dashed arrows). The red and blue curves represent the electronic bands with opposite orbital angular momenta.^37^

We start by examining the optical properties of α-FAPbI_3_ deposited on graphene/Si–SiO_2_ (hereafter referred to as graphene substrate) and Si–SiO_2_ (hereafter referred to as SiO_2_ substrate). Figure 1 shows the optical characterization and an illustrative schematic of the electronic band structure that is expected to appear due to the growth of the perovskite on the graphene layer. The figure is complemented by conceptual representation of lattice distortions induced by the interaction between perovskite and graphene (Figure 1a). First, we measured steady-state photoluminescence (PL). The as-grown layer on SiO_2_ exhibited remarkably strong PL (Figure 1b, purple), which was quenched by nearly 80% when deposited on single-layer CVD graphene (Figure 1b, light blue). We attribute this effect to the nonradiative recombination process and efficient charge transfer due to the built-in electric field established upon contact of two materials during perovskite deposition. The emission maximum of the perovskite film on SiO_2_ is located at 794 nm. The PL maximum on graphene/perovskite devices showed a slight redshift by ∼8 meV of the perovskite interband optical transition and corresponding emission maximum at 798 nm. We attribute the observed redshift primarily to strain in the crystal lattice induced by the interaction with graphene, which affects the electronic levels of the perovskite, as will be discussed later.

To gain deeper insight into the photogenerated charge carrier dynamics and carrier exchange between perovskite and graphene, we performed time-correlated single photon counting (TCSPC) measurements for perovskite on SiO_2_ and graphene (shown in Figure 1c,d, respectively). The fitting of the obtained data showed monoexponential decay for the perovskite on SiO_2_ (τ^pero^ = 23.6 ns) and biexponential decay for graphene samples. The two components for the perovskite on graphene (Figure 1d) showed much shorter lifetimes: τ_1_^comp^ = 2.5 ns (2% contribution) and τ_2_^comp^ = 0.9 ns (98% contribution). We interpret the kinetics of τ^pero^ as a decay component resulting from the second-order radiative carrier recombination.^50^ For the composite heterostructure, we attribute both components to the second-order radiative recombination similar to the decay in the perovskite film on SiO_2_, which is however affected by the formed heterointerface between the perovskite and graphene. It is commonly accepted that recombination kinetics in perovskite semiconductors follows mixed second/first-order rate law where the decay results from bimolecular recombination at higher charge carrier densities and trap-mediated (also known as Shockley-Read-Hall) recombination regime at lower densities.^50^ However, our TCSPC findings on graphene reveal indications of recombination through nonallowed transitions, resembling an indirect band gap semiconductor. One of the possible explanations might include influences of Rashba spin–orbit coupling or a mixture of indirect and direct band gap states, as pointed out in published studies.^33,51^ The existence of an indirect band gap and spin-split electronic bands in the heterojunction plays an important role in suppressing intrinsic radiative recombination channels. This is consistent with the spectral dominance of defect-state-induced emission, as evidenced by the perovskite PL redshift observed on graphene (Figure 1b). The formation of the effective built-in potential in combination with the Rashba term provides a mechanism for charge carrier separation. This mechanism allows the electrons from the perovskite film to be transferred to the graphene (prior to illumination). However, upon excitation, it triggers a migration of the photogenerated holes to the graphene layer, resulting in the strongly quenched PL emission.^52^

We also studied both samples using the polarized steady-state PL. Through circularly polarized excitation, we observed (Figure 1e) spin splitting in the electronic structure of the graphene/perovskite composite heterostructure, providing a higher PL quantum yield for left-handed (LCP or σ^–^) than right-handed (RCP or σ^+^) polarization. The typical polarized PL difference under 5 μW power (corresponding to 2.54 × 10^3^ W cm^–2^ irradiance in a confocal regime) of 532 nm excitation was found to be 18%. The anisotropy factor *g*_CPL_, given by the equation *g*_CPL_ = 2(*I*_σ–_ – *I*_σ+_)/(*I*_σ–_ + *I*_σ+_), where *I*_σ–_ and *I*_σ+_ represent the intensities of σ^–^ and σ^+^ circularly polarized emissions, was determined to be 0.35. This value is comparable or even superior to the most of published data on chiral perovskite devices.^29^ We provide a comprehensive comparison of *g*_CPL_ and *g*_ph_ values reported for chiral nanomaterials, including perovskite devices, in Table S1. On the contrary, for the α-FAPbI_3_ film on the SiO_2_ substrate without graphene, the *g*_CPL_ typically reached a value of only 0.02–0.03 (Figure S1). Hence, we obtain a PL anisotropy more than an order of magnitude higher for the graphene/perovskite composite heterostructure, suggesting more pronounced electronic band splitting in this case. Furthermore, we found a 3.91 meV shift in the PL maxima for the σ^–^ and σ^+^ excitations, which was not observed for the α-FAPbI_3_ film on SiO_2_ (Figure S1). Figure 1f schematically depicts the expected band structure of the perovskite, which features Rashba-type splitting with broken spin degeneracy manifested as the electronic bands, with opposite spin textures separated along the momentum and energy axes simultaneously. Both the valence band (VB) and the conduction band (CB) are formed by the hybridization of Pb and X orbitals,^53^ suggesting distorted inversion symmetry in the [PbX_6_]^4–^ cage of the perovskite. We hypothesize that specific pushing and pulling forces, arising from the interaction between the perovskite sublattices (iodine and lead) and graphene, lead to charge transfer at the interface during the film deposition. This concept is supported by the theoretical study,^49^ explaining it through electron depletion beneath the atoms of iodine sublattice and electron accumulation beneath the lead sublattice. These forces can be understood in terms of the electronegativity of corresponding atoms, e.g., χ(C) = 2.55, χ(I) = 2.66, χ(Pb) = 2.33. As a result, the perovskite lattice becomes distorted in a specific manner due to chemical interactions during film growth, which in consequence allows for enhanced LCP sensitivity. Here we speculate that different perovskite composition may lead to different preferential crystal orientations, potentially offering a pathway to enhanced polarization sensitivity with a particular orientation of the electric field vector. However, at this stage of understanding of such complicated systems, it is challenging to establish a general rule applicable to other combinations of perovskite and conductive substrates.

**Figure 2:**
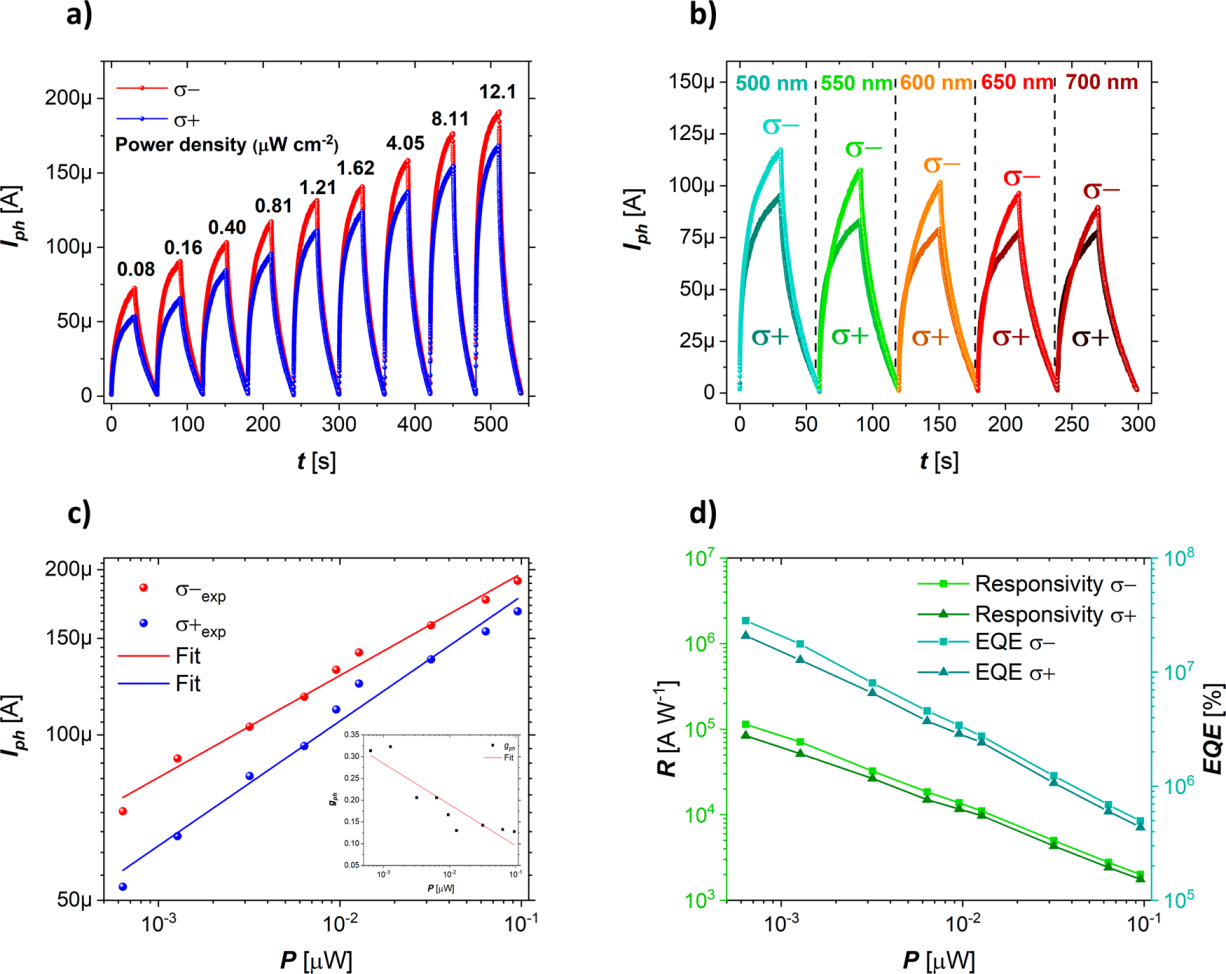
Manifestation of spin photoconductive effects in a graphene/α-FAPbI_3_ device observed using photocurrent measurement. (a) Power-dependent response of spin-polarized photocurrents measured using left (σ^–^, red spheres) and right (σ^+^, blue spheres) circularly polarized 500 ± 5 nm laser and a fixed *V*_DS_ of 0.5 V. (b) Wavelength-dependent response of photocurrents measured on the same sample in the range 500–700 nm with 0.81 μW cm^–2^ fluence for each excitation energy. (c) Photocurrents subtracted from power-dependent measurements and fitted according to a power law. (d) Responsivity (R) and external quantum efficiency (EQE) calculated for σ^–^ (squares) and σ^+^ (triangles) excitation.

Let us now turn to the electrical properties. First, we studied the devices’ optoelectronic performance through a power-dependent photocurrent (*I*_ph_) measurement using both circularly polarized excitations at a fixed wavelength of 500 ± 5 nm and a constant source–drain voltage (*V*_DS_) of 0.5 V. The setup is described in the Methods section. The device found a high net photocurrent yield in the microampere range, shown in Figure 2a, with the milliampere dark current produced by graphene subtracted for clarity. The current–voltage characteristics are available in Figure S2. The obtained photocurrent is positive, suggesting that illumination induces hole-dominated carrier transport in the graphene channel under positive bias.^52^Figure 2a shows reproducible CPL sensitivity over a wide illumination power range. This effect can be explained within the framework of SPEs. The optically generated spin-polarized exciton is separated at the materials’ junction, producing spin photocurrents in opposite directions. This manifests as a higher or lower photocurrent yield depending on the helicity of the excitation, as schematically shown in Figure 1f.

Using a fixed optical power density (∼0.81 μW cm^–2^), but otherwise under the same experimental conditions, we observe an identical effect of CPL sensitivity for a range of visible photon energies (Figure 2b). The measured photocurrent decreases monotonically toward low photon energies. The effect of CPL sensitivity persists up to 700 nm, where we observe a decrease in the *g*_ph_ anisotropy factor. We ascribe this to the proximity of the photon energy to the absorption band edge of α-FAPbI_3_ film on graphene. Hence, the effect is observed with photon energies above the band gap of the perovskite. The observation can be tentatively explained based on the momentum-dependent shift in the electronic bands (Figure 1f) due to the presence of the Rashba field.^35,37^ While it is plausible that CPL sensitivity could be enhanced at shorter wavelengths due to higher photon energy—potentially leading to more efficient excitation and greater anisotropy in the photocurrent—the actual photocurrent response is highly dependent on the dynamics of the excited states.^52,54^ This interaction with higher photon energies could alter the photocurrent behavior, possibly resulting in varying CPL sensitivity.

Figure 2c shows the measured power-dependent circularly polarized photoresponse fitted to a power law. The dependence of *I*_ph_ on power density exhibit an exponential profile (here shown in the logarithmic scale). Several factors could contribute to the observed nonlinearity in this relationship. For example, at higher light intensities, increased carrier generation can lead to enhanced recombination processes, such as bimolecular or Auger recombination. Under these conditions, the polycrystalline perovskite material may also develop trap states, which capture and release charge carriers in a nonlinear manner. Additionally, it is common for low-dimensional photodetectors to exhibit nonlinear behavior due to the photogating effect, where photogenerated charge carriers modulate the conductivity of the channel. While it is challenging to isolate individual contributions, these processes can collectively result in a nonlinear response in photocurrent generation, which will be consequently reflected in higher uncertainty of the photocurrent anisotropy factor, *g*_ph_. The variation in the obtained *g*_ph_ factor can be seen in the inset of Figure 2c. We observe an increase in *g*_ph_, reaching a value of 0.32 in the low fluence regime. We obtained a similar *g*_ph_ value as in the studies based on chiral perovskite sensors; however, in our case, no chiral molecule is present in the structure. This suggests an efficient transfer of the optically induced unbalanced spin population in the achiral perovskite film, which produces spin-polarized photocurrents available at the electrical read-out provided by the graphene channel. The responsivity *R = I*_ph_/*P*, where *P* is the illumination power, determined for σ^–^ excitation at the lowest optical power density of 80 nW cm^–2^, is 1.1 × 10^5^ A W^–1^, whereas σ^+^ excitation at the same power yielded a value of 8.3 × 10^4^ A W^–1^, as shown in Figure 2d. The photon-to-charge conversion using σ^–^ excitation is 1.3 times more efficient.

The high responsivity of the prepared devices can be attributed to the photoconductive gain mechanism available through the photogating effect.^55^ The photoexcited holes are transferred to the graphene, changing its Fermi level and producing a photocurrent, while negatively charged electrons are left in the perovskite film. As long as electrons remain trapped in the perovskite, holes in the graphene channel are recirculated in the circuit multiple times, allowing for >1 quantum efficiency. The photogenerated electrons trapped in the perovskite film create an additional electric field, which acts against the built-in electric field established by the alignment of the materials’ Fermi levels in thermal equilibrium (Figure S3). However, when the power density is higher than the absorption saturation limit, the photogating effect creates an electric field high enough to substantially reduce the built-in electric field, which consequently reduces the efficiency of exciton separation in the junction. The strength of this opposing field depends not only on the power but also on the polarization of the excitation photon, giving rise to a lower or higher amount of photocurrent.^52^ Both PL and photocurrent measurements suggest that the optical selection rules prefer spin-polarized excitons generated by photons with σ^–^ helicity. For both σ^–^ and σ^+^ excitations, the carriers with a lifetime >1 ns are involved in the photocurrent generation and photogating effect, while carriers with a lifetime <1 ns recombine.

Under a low excitation power, the electrons in the perovskite layer are well separated from the photogenerated holes, supporting strong carrier rectification in the circuit. In contrast, a higher power induces a denser population of photoexcited carriers, which reduces the separation between the trapped electrons and the mobile holes, thus increasing the probability of their recombination. This recombination of carriers at the higher illumination power reduces the responsivity as well as the trapped electron lifetime.^52,54^ A similar trend is observed when the external quantum efficiency (EQE) is plotted against the illumination power *P* (Figure 2d): σ^–^ excitation yields 2.8 × 10^7^% at the lowest incident power, while σ^+^ excitation provides 2.1 × 10^7^% of apparent photon-to-electron conversion efficiency. The *R* and EQE values can be further improved by increasing V_DS_ in the channel by applying a gate voltage.^55^

The obtained photocurrents were fitted to a double-exponential curve, yielding rise time constants of *t*_1_ = 870 ± 20 ms and *t*_2_ = 180 ± 10 ms. These times are consistent with published values for bulk perovskite-based graphene photodetectors.^18^ Details on the fitting of the photocurrent rise and fall times can be found in Figure S4. The larger time constant *t*_1_ is mainly due to the photoexcited carriers traveling through the bulk perovskite crystal, while the shorter *t*_2_ can be ascribed to the carriers originating from the area closer to the heterojunction. There seems to be a contradiction between the photocurrent measurement, which shows a response time of ∼870 ms, and the TCSPC measurement, which suggests that all charge carriers with a lifetime >1 ns contribute to the photocurrent. This effect can be explained by a combination of two factors: (1) a variation in light penetration depth with illumination power, and (2) the actual thickness of the perovskite film. At a higher illumination power, the greater penetration depth leads to more uniform excitation throughout a larger volume of the perovskite film. Conversely, lower power regimes result in shorter penetration depths, leading to exciton generation primarily in the top outermost thin layer of the perovskite crystallites. As a result, charge carriers must travel through the film volume up to the graphene layer, influenced by the built-in electric field, which may contribute to the lengthening of the overall response time. Additionally, polycrystalline solution-processed perovskites have grain boundaries with intrinsic barriers (shallow traps) that can affect the mobility of traveling carriers.^56^

**Figure 3:**
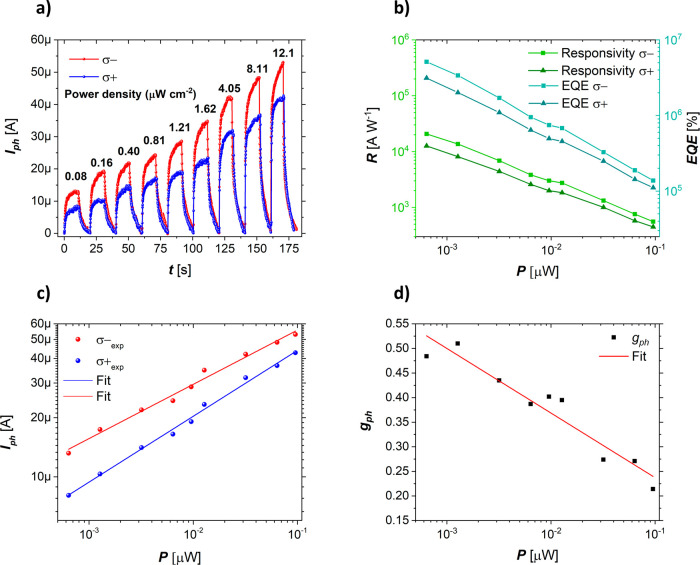
Spin photoconductive effect observed in the graphene/α-FAPbI_3_ device through polarized photocurrent measurement in the thin perovskite film. (a) Power-dependent response of spin-polarized photocurrents measured using left (σ^–^, red spheres) and right (σ^+^, blue spheres) circularly polarized 500 ± 5 nm laser and a fixed *V*_DS_ of 0.5 V. (b) Calculated responsivity and external quantum efficiency for the thin perovskite film on graphene obtained using σ^–^ (squares) and σ^+^ (triangles) excitations. (c) Photocurrents subtracted from power-dependent measurements and fitted to a power law. (d) The variation of the photocurrent anisotropy factor. The red line is a guide for the eye.

As we assumed that the observed CPL effects originate from the impaired centrosymmetry in the first few perovskite layers just above the graphene, we attempted to emphasize the impact of inversion symmetry-breaking distortions by further reducing the total thickness of the deposited layer and decreasing the amount of bulk perovskite. The prepared sample was characterized (Figure S5) using atomic force microscopy (AFM). By again performing photocurrent measurements using CPL excitations, we obtained even greater CPL sensitivity (Figure 3).

As expected, we achieved a lower overall photocurrent yield (Figure 3a) due to the reduced amount of bulk photoactive perovskite; however, the photocurrent anisotropy was improved, with a 1.5 times higher *g*_ph_ value of 0.51 in a low-power regime (Figure 3c,d). To the best of our knowledge, this is the record setting *g*_ph_ value that has been obtained in achiral perovskite-based devices without the use of additional organic-to-inorganic chirality transfer or applied external fields (Table S1).^57−59^ The same trend can be observed in the plots of *R* and EQE against *P* (Figure 3b). For these samples, we obtained responsivities of 2.1 × 10^4^ A W^–1^ and 1.2 × 10^4^ A W^–1^ for σ^–^ and σ^+^ excitation, respectively, indicating that the major portion of the device performance originates in the heterojunction region. The lower overall responsivity, in this case, can be explained by its tight relation to the photocurrent yield and the trade-off between the light absorption and the charge transfer efficiency. Compared with devices based on thicker perovskite layers, the transient photocurrent saturation time of the thinner devices is shorter for both left- and right-handed polarizations. The saturation of the photocurrent occurs more rapidly when using σ^+^ excitation compared to σ^–^, suggesting fewer available transitions for 500 nm σ^+^ polarized light. In contrast, the σ^–^ excitation exhibits a greater number of available transitions at higher power density. Such an effect was not observed in the thicker film. We attribute this to the more dominant graphene-induced generation of the Rashba field in thinner perovskite layers. We also exploited steady-state PL to examine how the charge transfer was improved due to the reduced absorber thickness. However, the composite with thin perovskite film did not show any PL, which suggested complete quenching due to even more efficient charge transfer (Figure S8).

## Conclusions

In summary, our study presents a promising perspective in researching spin-associated optoelectronic devices based on solution-processed perovskite semiconductors. We observed that graphene can serve as a template for perovskite growth, leading to local inversion symmetry breaking within the heterojunction region, but otherwise sustaining bulk perovskite crystal properties. By carefully engineering the interface, we achieved significant circularly polarized PL anisotropy and managed to transfer the effect to an electrical read-out represented by the spin-polarized photocurrent anisotropy. Furthermore, we demonstrated how perovskite growth on graphene effectively suppresses radiative recombination and enables efficient charge carrier extraction, which is crucial for advancing perovskite optoelectronics. The templating effect is likely to be observed in other achiral perovskite compositions grown on carbon-based materials and semiconducting substrates. Of high importance are the electrostatic forces between adjacent atoms of perovskite and the substrate material, as well as perovskite’s crystal lattice orientation. Additionally, templated low-dimensional perovskites may exhibit enhanced effects due to the combination of interface and bulk Rashba terms. The flexibility in tuning perovskites’ properties and the rational choice of the channel material provide an elegant way to manipulate electron spin by adjusting the Rashba effect in switchable spin devices. Though the idea of adding new functionality through the optimization of SOC-related effects is appealing, many questions remain unanswered, requiring further research.

## Methods

### Graphene Preparation

The CVD graphene was synthesized on a Cu foil (99.8%, Alfa Aesar) using a mixture of CH_4_ (1 sccm) and H_2_ (50 sccm) in a quartz tube reactor at 1000 °C and 350 mTorr pressure. Before the methane injection, the Cu foil was annealed for 40 min in an H_2_ atmosphere. The graphene samples were transferred by the PMMA protocol onto 300 nm Si–SiO_2_ wafers (MicroChemicals GmbH) and annealed in an Ar/H_2_ (3:1) atmosphere for 2 h at 250 °C. The prepared samples were characterized by Raman spectroscopy. A typical Raman spectrum of graphene on SiO_2_ substrate before α-FAPbI_3_ deposition can be found in Figure S9. The electrodes were thermally evaporated through a shadow mask on top of the transferred and annealed graphene. Each studied sample consisted of a matrix of 20 devices with a 10 μm channel gap.

### Perovskite Film Preparation

The state-of-the-art α-FAPbI_3_ films on graphene were prepared in a low-humidity (RH ∼ 10%) atmosphere and contained 0.8 M of FAI and PbI_2_ with an added 35–40 mol % of MACl in anhydrous dimethylformamide/dimethyl sulfoxide (4:1, v/v) to achieve the desired composition, α-FAPbI_3_ (5% PbI_2_ excess). The perovskite solution was spin-coated in a one-step program at 5000 rpm. Subsequently, 270 μL of chlorobenzene was dropped on the spinning substrate. The films were then annealed at 150 °C for 35–40 min.^60^ To ensure that the black phase of α-FAPbI_3_ was obtained, we performed an XRD experiment, in which no additional peak associated with the yellow phase was observed (Figure S6a). Furthermore, surface morphology images were obtained using scanning electron microscopy (SEM) to gain insight into the coverage of the film on the substrate. These images clearly showed that the graphene was fully covered and that the perovskite film was uniform and highly crystalline, with similar grain domain sizes of hundreds of nanometers (Figure S6b).

### Device Characterization

The morphology of both thicker and thinner α-FAPbI_3_ film on graphene was studied on a series of devices by Bruker Icon AFM in peak force mode, using SiN tips with a 2 nm nominal radius. AFM images, RMS roughness, and other statistical quantities are available in Figures S7 and S5 for the thicker and thinner films, respectively.

The steady-state PL spectra were collected using a WITec Alpha 300R spectrometer equipped with 1800 gr/mm grating and a continuous-wave 532 nm laser in a confocal regime. For polarized PL measurements, two sets of polarizing plates were applied. The first set of λ/2 and λ/4 plates were used to choose the excitation polarization. The angle of the emission polarizing plates matched the excitation polarization.

TCSPC mapping was performed on a home-built confocal microscope. The excitation source (pulsed diode laser LDH-470, controlled by Sepia II, PicoQuant) operated at 2.64 eV pulsed excitation at a laser power of 5 μW, a repetition frequency of 5 MHz, and a pulse duration of 70 ps. The excitation light was focused into the sample through an air objective (Olympus, 0.65 NA, 40×). The excitation and emission light were separated by a Z473 dichroic mirror (Chroma). The emitted light was further guided through a 50 μm pinhole into the detection channel, which was equipped with an LP580 long-pass filter (Chroma) and an MPD detector (PDM). The photons were registered using a Hydraharp 400 TCSPC module (PicoQuant). For graphene/perovskite composites, the acquisition time had to be set to longer values than for the perovskite film deposited on SiO_2_ substrate due to the high quenching efficiency caused by the presence of graphene. The photon arrival times considering the previous laser pulse were stored for each photon event, using ultrafast electronics in a time-tagged time-resolved (TTTR) recording mode. For spatially resolved lifetime measurements, fluorescence lifetime imaging microscopy (FLIM) images were acquired using a fast-FLIM approach, in which each pixel represents the average arrival time of the emitted photons. Finally, the excited state carrier lifetimes were obtained by analyzing the lifetime decays calculated from the regions of interest (on the perovskite deposited either on graphene or SiO_2_) using an iterative deconvolution process with PicoQuant Fluofit software on a multiexponential function convoluted with the instrument response function (IRF).

**Figure 4:**
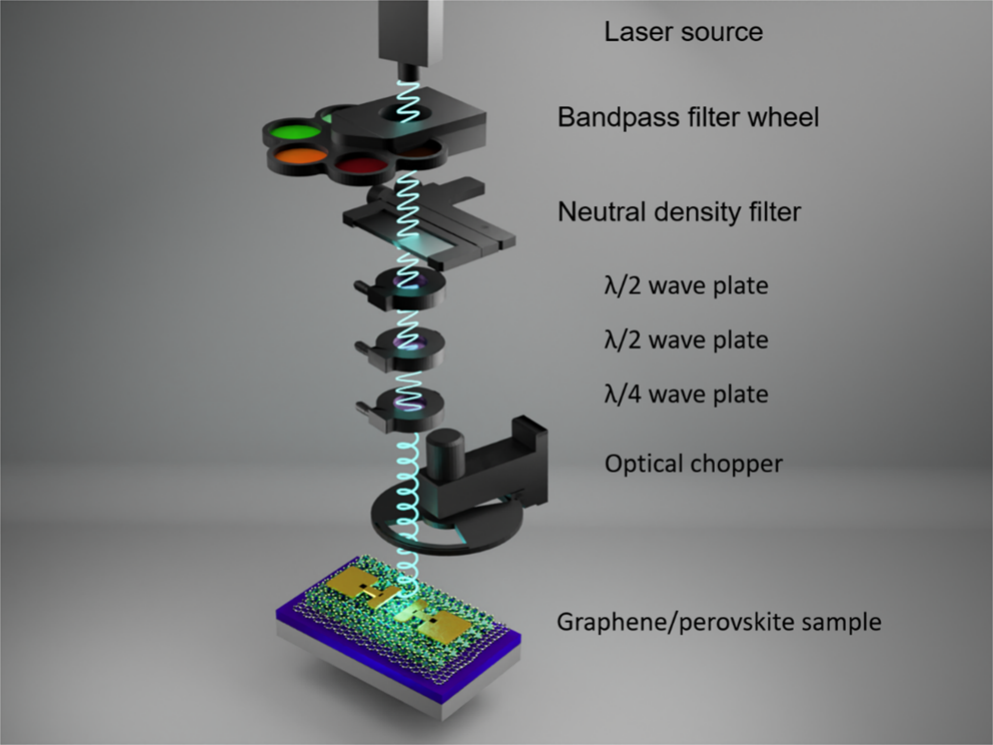
Arrangement and geometry of the optical path used for the polarized photocurrent measurement of the graphene/perovskite devices.

Electrical properties were obtained using a Keithley 2612B electrometer in a four-wire two-point probe setup. The photocurrent measurement was performed by illuminating the sample with a continuous-wave white laser (Coheras, NKT Photonics) and filtering with 10 nm bandpass filters, followed by a silver-coated neutral density filter and an optical chopper. The optical power meter was introduced into the optical path as needed. For manipulation of optical polarization, a set of polarizing plates (two λ/2 and one λ/4) was introduced in the optical path. An illustration of the setup is shown in Figure 4. By rotating the first λ/2 plate, we found the maximum intensity of the initial laser polarization and set the second λ/2 plate accordingly to obtain the maximum power for the desired linear polarization. Relative to the second λ/2 plate, we placed the λ/4 plate to obtain a circular polarization. To avoid any unwanted changes in light polarization within the optical path, we excluded mirrors and conducted all measurements with an excitation incidence angle perpendicular to the plane of the sample. Prior to the steady-state PL and photocurrent measurements, the light sources’ polarization was precisely selected within the Poincaré sphere using a PAX1000VIS polarimeter (Thorlabs).

## Supporting Information Available

Figure S1: PL data of thick α-FAPbI_3_ film on SiO_2_ without graphene. Figure S2: Current–voltage characteristics of graphene/α-FAPbI_3_ device. Figure S3: Band diagram of graphene/α-FAPbI_3_. Figure S4: Photocurrent fitting. Figure S5: AFM of thin film sample, height distribution. Figure S6: X-ray diffraction data, electron microscopy data. Figure S7: AFM of thick film sample, height profile. Figure S8: PL of thin α-FAPbI_3_ film on SiO_2_ and graphene. Figure S9: Raman spectrum of graphene prior to perovskite deposition. Table S1: Comparison of performance characteristics in terms of anisotropy factors. Table S2: Fitting parameters of the rise time. Table S3: Fitting parameters of the decay time (PDF)
