# Supplementary material for: Graphene-Templated Achiral Hybrid Perovskite for Circularly Polarized Light Sensing
Source: ACS Appl Mater Interfaces. 2024 Sep 19;16(39):52789–98. doi: 10.1021/acsami.4c10289 (PMC11450682; doi:10.1021/acsami.4c10289)
Supplement: Supplementary file 1 — am4c10289_si_001.pdf [file am4c10289_si_001.pdf]

Supporting Information

Graphene-Templated Achiral Hybrid Perovskite for Circularly Polarized Light Sensing

Oleksandr Volochanskyi[1,2], Golam Haider[3], Essa A. Alharbi[4,5], George Kakavelakis[5,6], Martin Mergl[1], Mukesh Kumar Thakur[1], Anurag Krishna[5], Michael Graetzel[5], Martin Kalbáč[1]**

[1] J. Heyrovsky Institute of Physical Chemistry of the Czech Academy of Sciences, Department of Low-dimensional Systems, Dolejškova 2155/3, 18223, Prague, Czech Republic

[2] University of Chemistry and Technology in Prague, Faculty of Chemical Engineering, Department of Physical Chemistry, Technická 5, 14200, Prague, Czech Republic

[3] Current address: Institute for Metallic Materials, Leibniz Institute for Solid State and Materials Research Dresden, Helmholtzstraße 20, 01069 Dresden, Germany

[4] Microelectronics and Semiconductors Institute, King Abdulaziz City for Science and Technology (KACST), Riyadh 11442, Saudi Arabia

[5] École Polytechnique Fedérale du Lausanne, Laboratory of Photonics and Interfaces, Station 6, Lausanne 1015, Switzerland

[6] Department of Electronic Engineering, School of Engineering, Hellenic Mediterranean University, Romanou 3, Chalepa, GR-73100, Chania, Crete, Greece.

KEYWORDS: chirality, optical helicity sensing, Rashba splitting, graphene, perovskite, photodetector

*Corresponding author email: g.haider@ifw-dresden.de and martin.kalbac@jh-inst.cas.cz

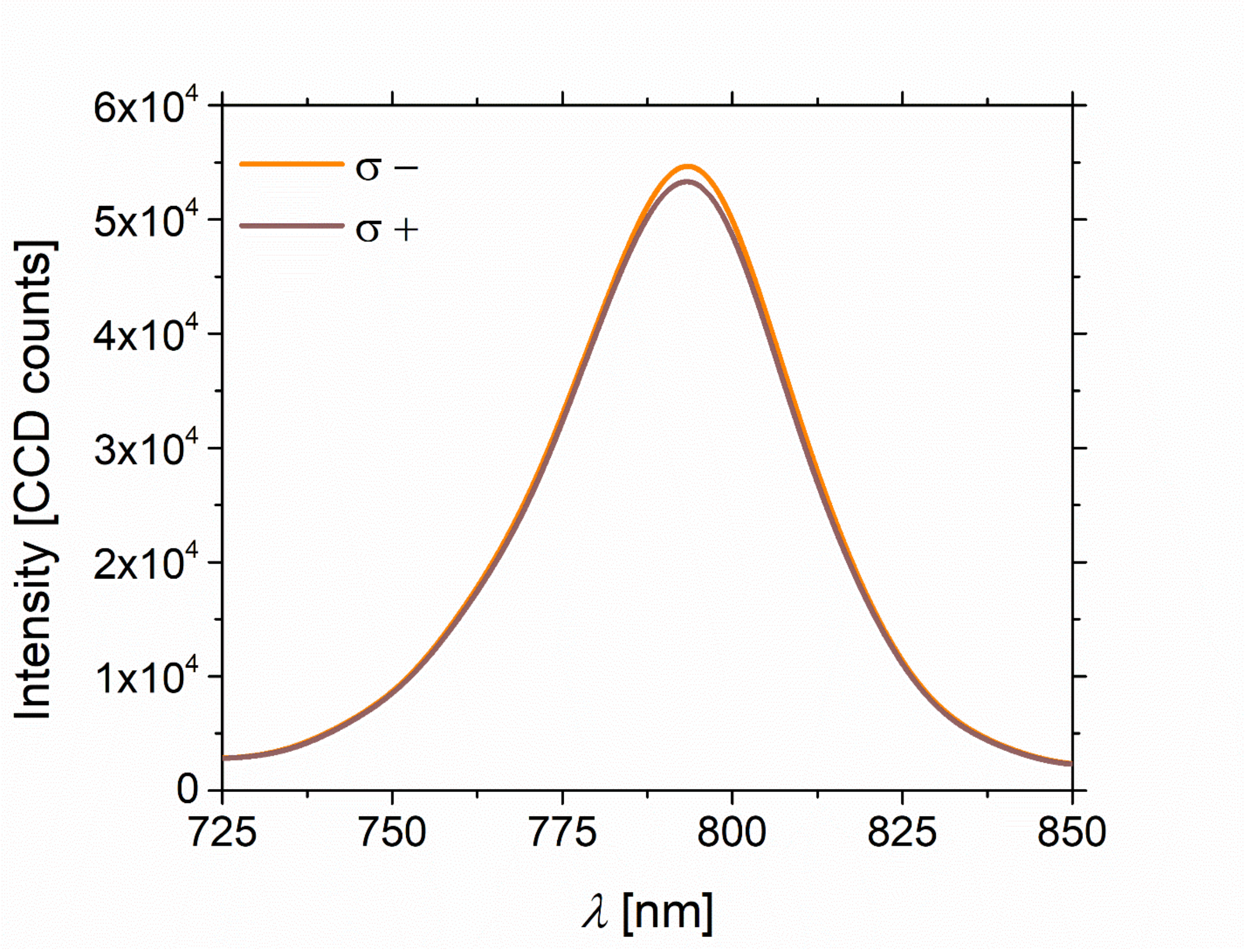

Figure S1. An average circularly polarized PL spectra of α-$FAPbI_3$ film deposited on SiO_2 substrate without graphene, collected in a confocal regime. The σ- (orange) and σ+ (brown) curves correspond to left and right circular excitation, respectively.

Figure S1 shows the obtained circularly polarized PL response of α-$FAPbI_3$ film deposited on SiO_2 substrate using 2.54×10^3 W cm^{-2} of 532 nm laser. The calculated PL anisotropy factor (g_{PL}) is low (typically 0.02–0.03) compared to the g_{PL} at the graphene/perovskite heterostructure (0.35) under the same illumination power. It is worth noting that in the case of α-$FAPbI_3$ deposited on SiO_2 (no graphene), we have not observed a shift of the PL maxima for both circularly polarized excitations.

Table S1. Comparison of photoluminescence and photocurrent anisotropy factors.

**Material**	g_{CPL}	g_{ph}	**Reference**
α-$FAPbI_3$/SLG	0.35	0.32/0.51	This study
R/S [DMA]$PbCl_3$	/	0.296	10.1021/acs.nanolett.4c02125
R/S $[PEA]_2PbI_4$	/	0.13	10.1039/D3TC01534C
R/S $[MBA]_2PbI_{4(1-x)}Br_{4x}$	0.064	/	10.1038/s41467-022-31017-9
R/S $[PEA]_2PbI_4$	/	0.23	10.1021/acsnano.9b04437
R/S [PPA] $EAPbCl_4$	/	0.4	10.1002/anie.202307034
L/R $MAPbBr_3$	/	0.38/0.39	10.1038/s41557-023-01290-2
d(*l*)-Zn-HOIF NFs	0.0017	/	10.1038/s41467-023-43700-6
CityU-7/CityU-8	/	0.5	10.1002/adma.202306414

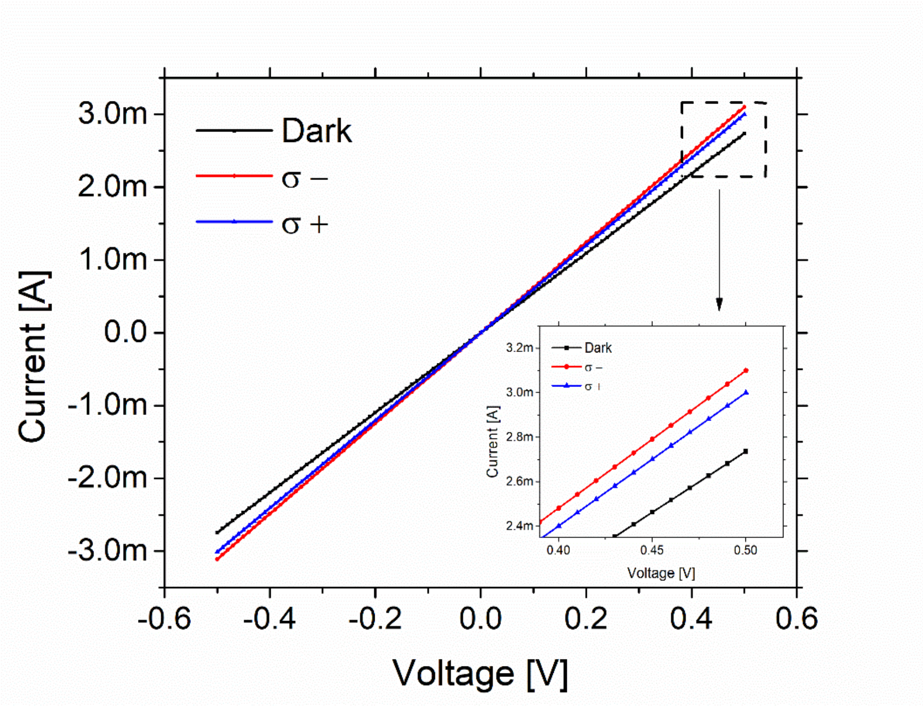

Figure S2. Current-voltage (IV) characteristic of the prepared graphene/α-$FAPbI_3$ device. The black line (squares) corresponds to the IV response in the dark. The red line (circles) corresponds to the IV characteristic under σ- excitation of 500 nm (162 μW cm-

[2]) laser illumination. The blue line (up triangles) corresponds to the IV characteristic under σ+ excitation of 500 nm (162 μW cm-[2]) laser.

The studied graphene/perovskite device shows linear current-voltage (IV) characteristics depicted in Figure S2. The architecture of the layered device suggests that the graphene acts as a charge collector, which only reflects the changes in the perovskite material under illumination. The σ- shows a higher photocurrent yield compared to σ+ excitation, consistent with PL and photocurrent measurements described in the main text. The inset shows a detail of current-voltage characteristics in the range of 0.4–0.5 V.

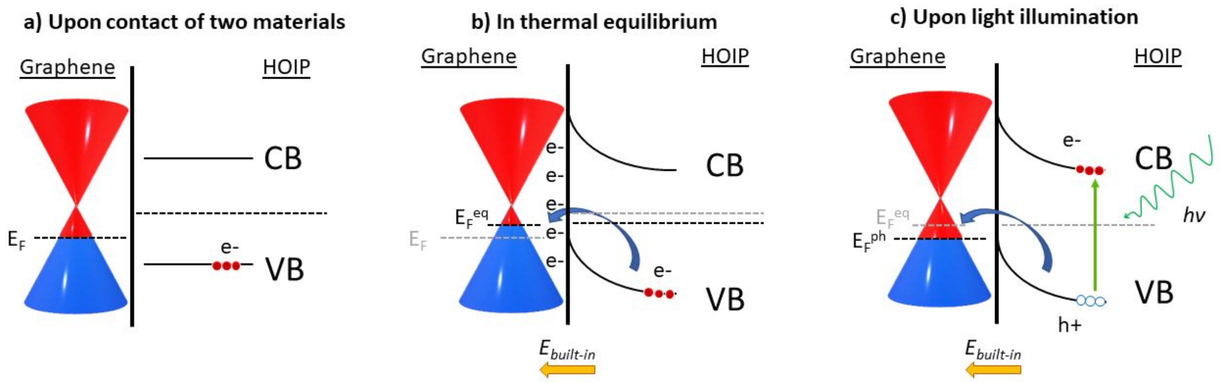

Figure S3. A schematic representation of band diagram in graphene/perovskite composite and the changes associated with a) before the formation of a surface junction and subsequent charge redistribution upon deposition of perovskite on the graphene layer; b) consequent alignment of corresponding Fermi levels (E_F^{eq}) and generation of a built-in electric field in thermal equilibrium; c) transfer of exciton-related holes from the valence band (VB) to the graphene, which gives rise to a photocurrent and simultaneous accumulation of exciton-related electrons in the perovskite conduction band (CB) in the general case of non-polarized light. The amount of generated photocurrent corresponds to the difference between E_F^{eq} and E_F^{ph}.

The optoelectronic operation can be understood through the band diagram of the device shown in Figure S3. The initial position of the Fermi level in single-layer graphene is dictated by the doping arising from the SiO_2 substrate, polymer residues from the transfer procedure, and the strain induced by the annealing process. [1, 2] It is known that transferred CVD graphene is p-doped, which is reflected by the position of E_F in Figure S3a). Also, hybrid perovskites are known to be p-type semiconductors. Therefore, an upward band bending is expected to appear in thermal equilibrium upon contact of two materials. [3] After the

perovskite deposition, the charge redistribution and Fermi levels alignment take place, upon which the electrons from the perovskite are transferred to the graphene, raising its Fermi level up to the position at thermal equilibrium in the dark E_F^{eq} Figure S3b). Owing to charge redistribution, the built-in electric field ($E_{built-in}$) is formed at the heterojunction. Upon light illumination (Figure S3c)), the photogenerated holes are transferred to graphene, while photogenerated electrons are trapped in the perovskite film.

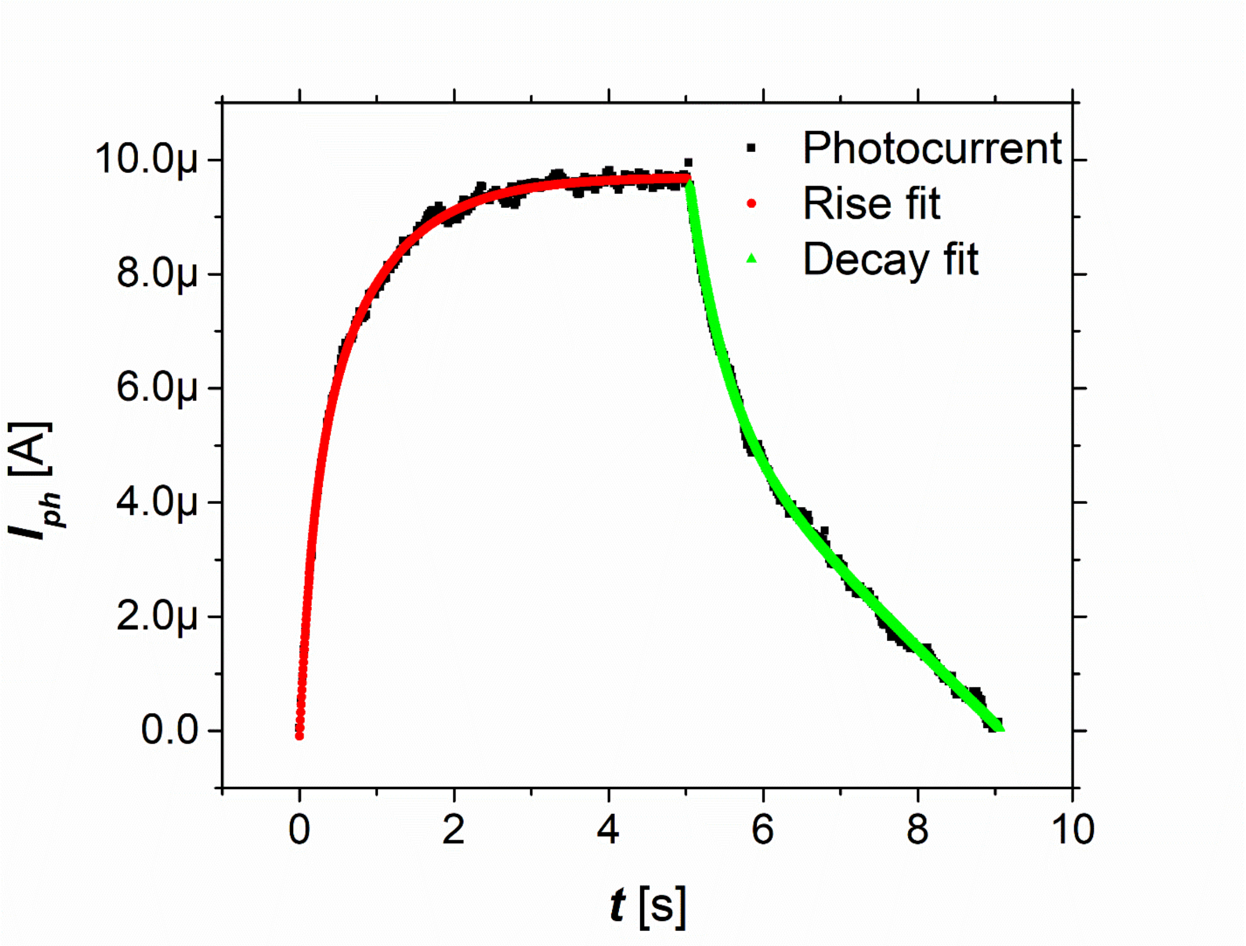

Figure S4. The fitting of the rise and decay time for the graphene/α-$FAPbI_3$ thin film device. The black squares correspond to the measured data, the red circles correspond to the fitting of the rise time, and the green up triangles correspond to the decay time fitting.

The obtained photocurrent (***I**$_{ph}$*) vs. time (***t***) curves were fitted using two different models (Figure S4). The Levenberg-Marquardt iteration algorithm was applied. The rise time was fitted according to the following equation:

$$y = A_1 \cdot exp\left(-\frac{x}{t_1}\right) + A_2 \cdot exp\left(-\frac{x}{t_2}\right) + y_0$$

where A_1 and A_2 corresponds to the amplitudes, t_1 and t_2 are the time constants and y_0 is the offset. The calculated values are summarized in the following Table S2:

Table S2. Summary of the calculated values of fitting parameters for the photocurrent rise time. SE is the standard error.

y_0		A_1		t_1		A_2		t_2		Statistics	
**Value**	**SE**	**Value**	**SE**	**Value**	**SE**	**Value**	**SE**	**Value**	**SE**	χ^2	R^2
3.878e-6	5.268e-9	-2.328e-6	6.643e-8	0.871	0.022	-1.585e-6	6.521e-8	0.179	0.010	1.527e-15	0.997

The decay time of the photocurrent was fitted according to the following equation:

$$y = p_1 \cdot exp\left(-\frac{x}{p_2}\right) + p_3 + p_4 \cdot x$$

where the p_1 and p_4 are the coefficients, p_3 is the offset, and p_2 is the unknown decay constant. The calculated values for the decay fitting are summarized in the following table:

Table S3. Summary of the calculated values of fitting parameters for the photocurrent decay time. SE is the standard error.

p_1		p_2		p_3		p_4		Statistics	
**Value**	**SE**	**Value**	**SE**	**Value**	**SE**	**Value**	**SE**	χ^2	R^2
1.676e-6	2.346e-8	0.502	0.016	2.147e-6	2.239e-8	-5.303e-7	7.279e-9	2.189e-15	0.998

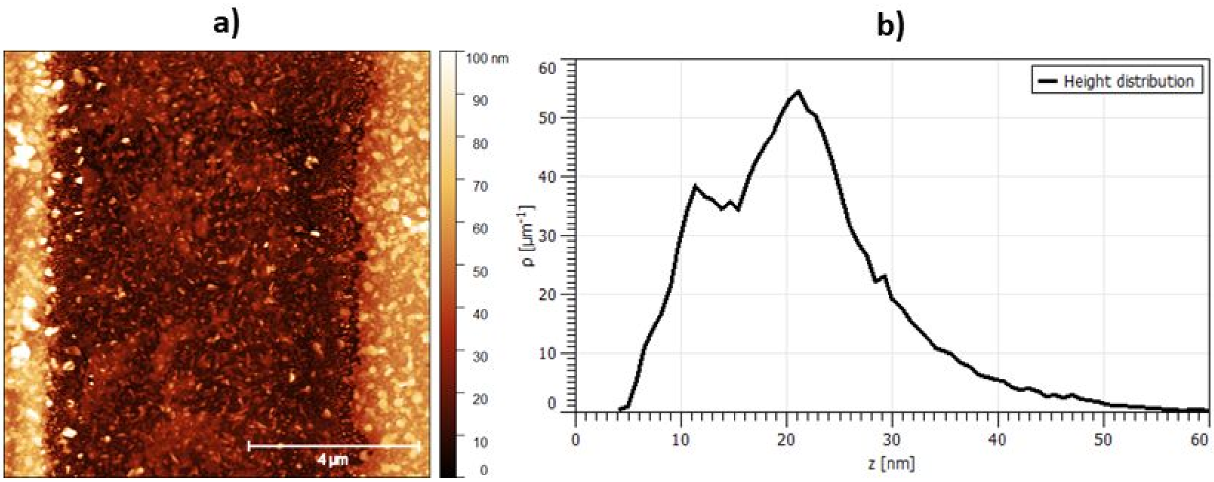

Figure S5. AFM characterization. a) A typical AFM scan of the gap between the electrodes in a graphene/thin α-$FAPbI_3$ film studied in the latter part of the main text; b) Subtracted height distribution of the perovskite crystallites within the gap of the device.

Figure S5a) shows a typical AFM scan of the graphene/α-$FAPbI_3$ thin perovskite device with electrodes on the left and right sides of the scan. The average height distribution of the perovskite crystallites is 21.1 nm (Figure S5b) having RMS roughness of 9.437 nm.

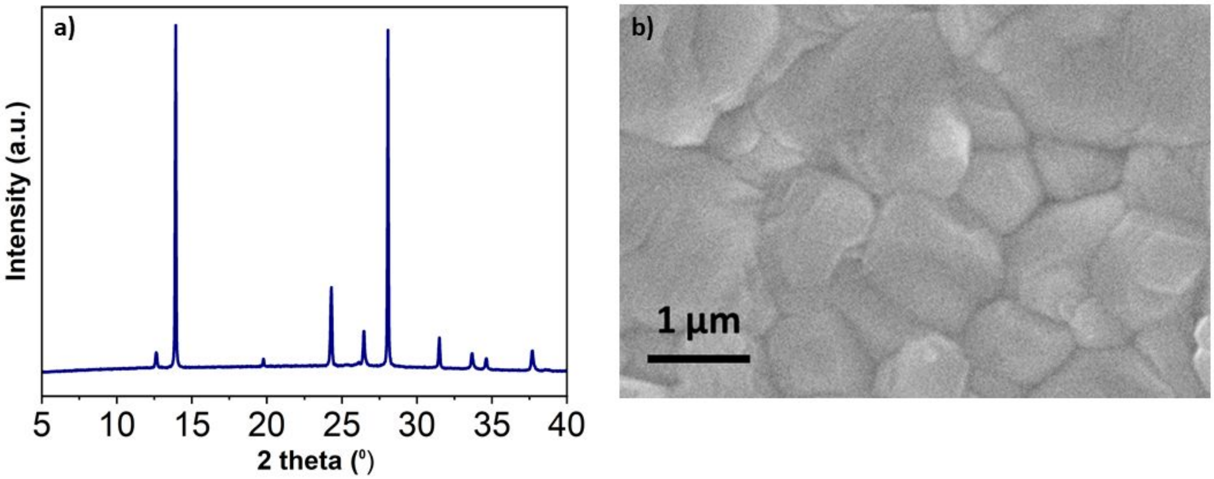

Figure S6. Characterization of the α-FAPbI3 hybrid perovskite film: a) XRD pattern, b) SEM image.

Figure S6a) shows the collected XRD pattern of the perovskite film deposited according to the conditions mentioned in the Methods part of the main text. The obtained pattern exhibits narrow signals of the cubic α-$FAPbI_3$ perovskite with a tiny amount of PbI2 [001], which can be found at $2\theta = 12.6°$. The two most

intense signals correspond to [111] and [222] cubic facets of the perovskite. Figure S6b) shows surface morphology obtained through SEM imaging with large perovskite crystal grains suggesting high-quality material.

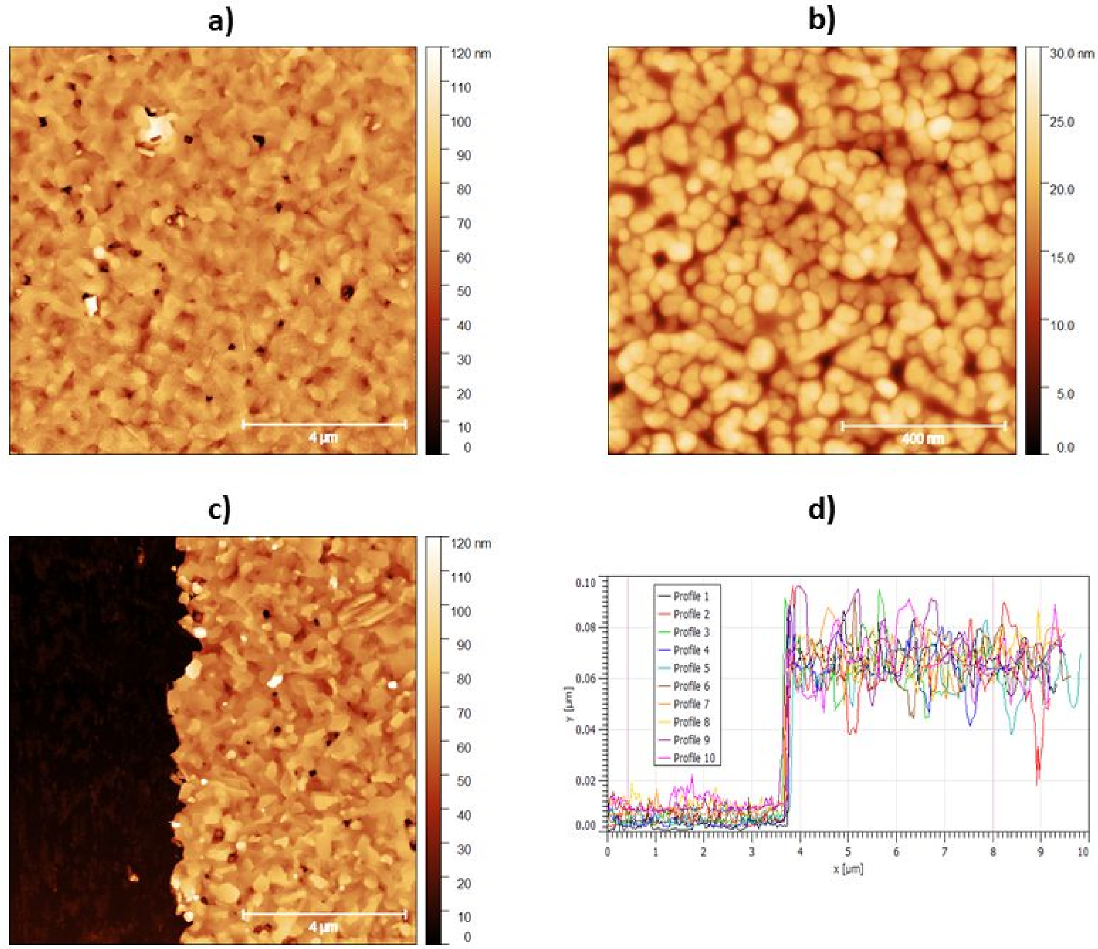

Figure S7. AFM characterization. a) AFM scan of the thicker α-$FAPbI_3$ film deposited on graphene and studied in the first part of the main text, b) detailed AFM scan, c) scratch in the perovskite layer made to estimate the thickness of the film, d) height profiles subtracted from c). Scan dimensions: 10x10 µm (a), c)) and 1x1 µm (b)).

Figure S7 shows collected AFM data: a) depicts the morphology of the prepared perovskite film with a small number of voids (dark spots) and a negligible number of peak heights (bright spots). The morphology of the film is reasonably flat for the solution-processed thin-film perovskite (RMS roughness is 9.245 nm). Figure S7b) shows the polycrystalline nature of the perovskite. Figure S7c) shows an intentionally made

scratch in the prepared film used for the estimation of the deposited film height. Figure S2d) shows 10 subtracted height profiles obtained from the AFM scan in Figure S7c). The average height corresponds to 68.3 ± 7.2 nm.

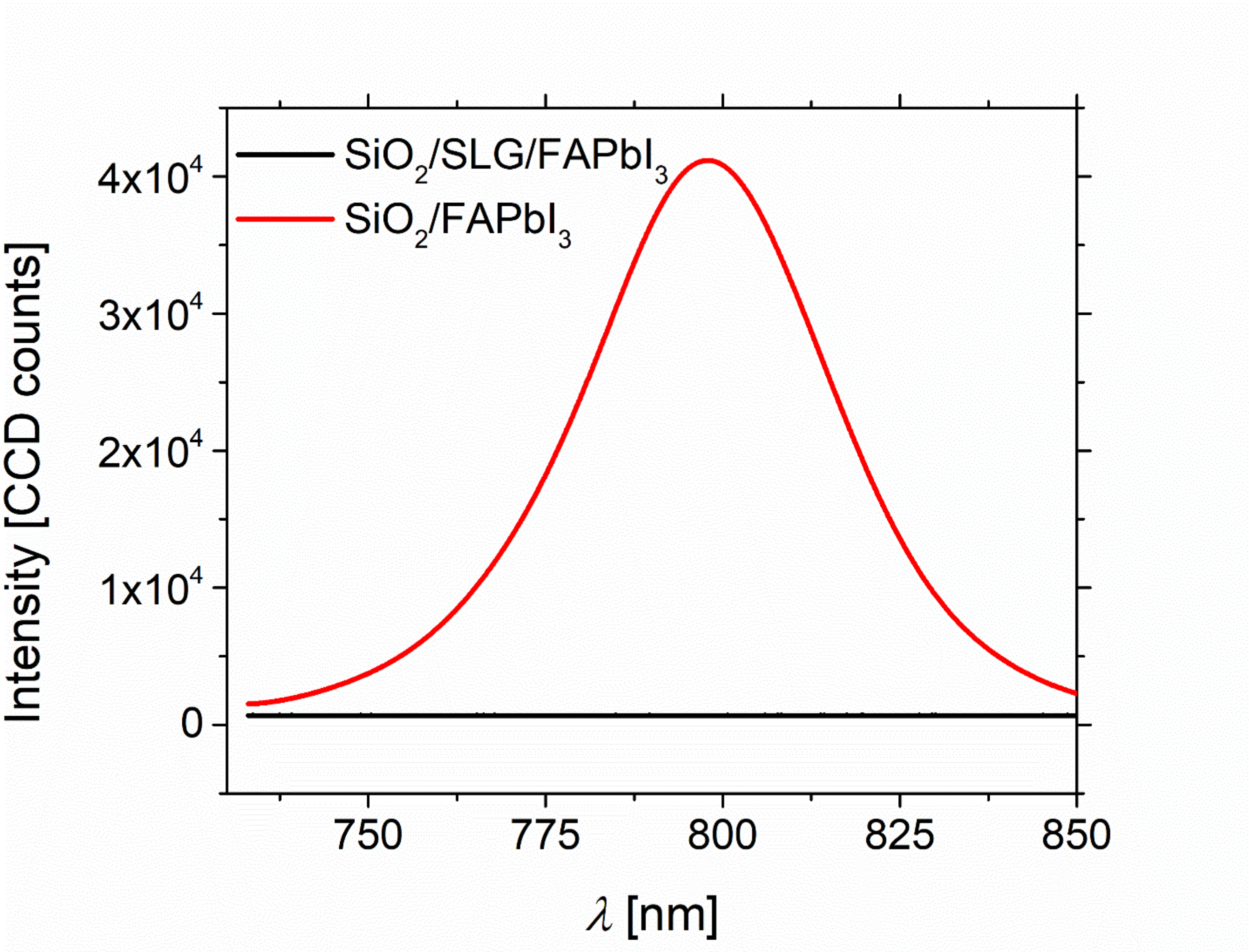

Figure S8. A comparison of PL spectra collected on graphene/α-$FAPbI_3$ thin perovskite composite (black) and α-$FAPbI_3$ thin film deposited on SiO_2 substrate (red).

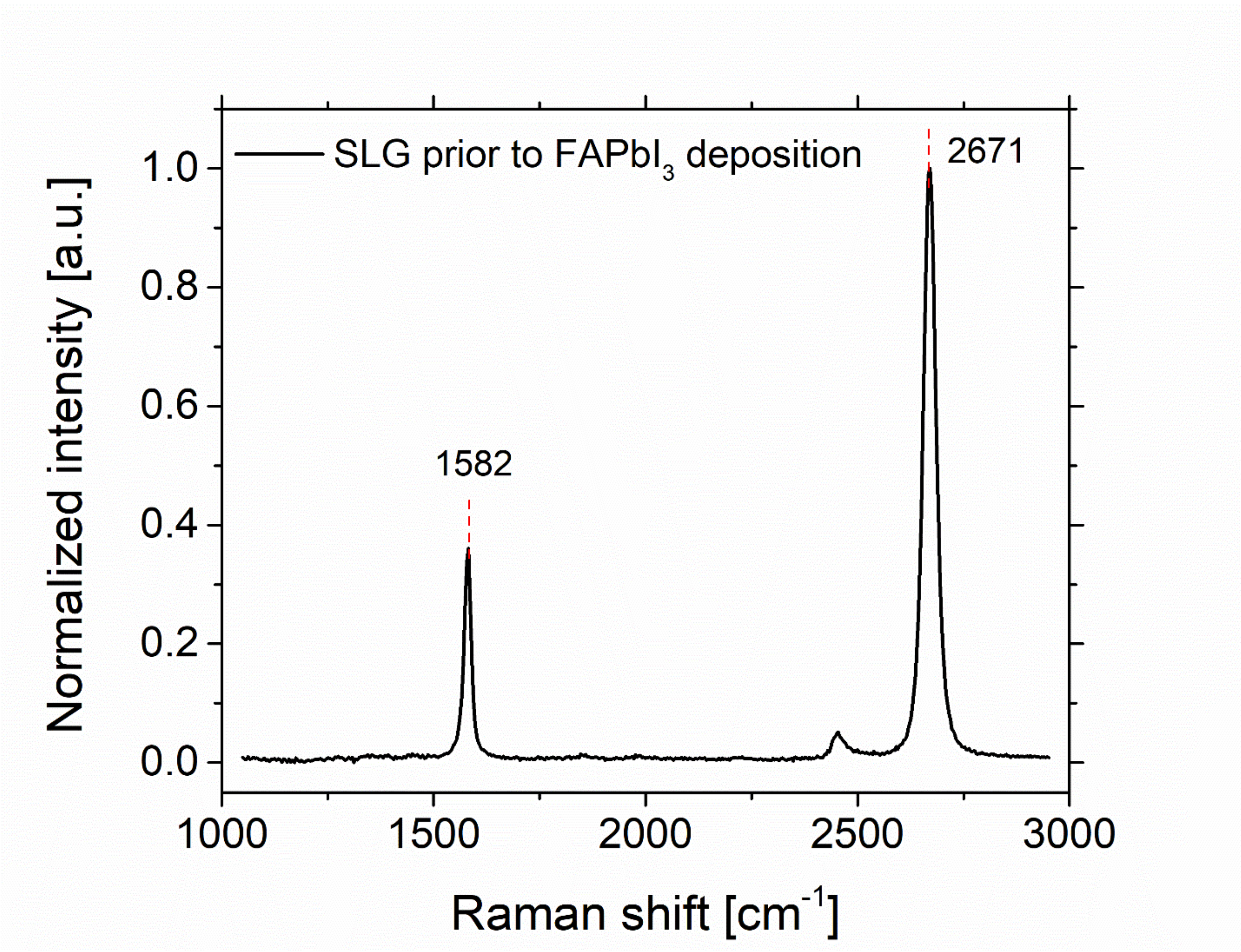

Figure S9. A typical Raman spectrum of single-layer graphene (SLG) before α-$FAPbI_3$ perovskite deposition. The data were collected using a 532 nm laser with 1 mW power.

Figure S9 shows a representative Raman spectrum of the graphene layer before perovskite deposition. The spectrum features prominent G and 2D peaks, characteristic of single-layer graphene. The G mode is located at 1582 cm^{-1} and the 2D mode is located at 2671 cm^{-1}. The 2D/G ratio is ~2.6 together with FWHM(G) = 18 cm^{-1} and FWHM (2D) = 25 cm^{-1}, and absence of the D mode around 1340 cm^{-1}, confirms the high quality of the synthesized monolayer graphene, which is essential for ensuring strong interaction with the perovskite film.

REFERENCES

(1) Costa, S. D.; Weis, J. E.; Frank, O.; Fridrichová, M.; Kalbac, M. Monitoring the doping of graphene on SiO2/Si substrates during the thermal annealing process. *RSC Adv.* **2016**, *6* (76), 72859-72864. DOI: 10.1039/C6RA10764H
(2) Kang, Y.-J.; Kang, J.; Chang, K. J. Electronic structure of graphene and doping effect on SiO2. *Phys. Rev. B* **2008**, *78* (11), 115404. DOI: 10.1103/PhysRevB.78.115404

(3) Wang, Y.; Zhang, Y.; Lu, Y.; Xu, W.; Mu, H.; Chen, C.; Qiao, H.; Song, J.; Li, S.; Sun, B.; et al. Hybrid Graphene–Perovskite Phototransistors with Ultrahigh Responsivity and Gain. *Adv. Opt. Mater.* **2015**, *3* (10), 1389-1396. DOI: 10.1002/adom.201500150
